# Supplementary figures and images for: pADP-ribosylation regulates the cytoplasmic localization, cleavage, and pro-apoptotic function of HuR
Source: Life Sci Alliance. 2024 Mar 27;7(6):e202302316. doi: 10.26508/lsa.202302316 (PMC10972696; doi:10.26508/lsa.202302316)

FigS1A

Representative blot for final figure

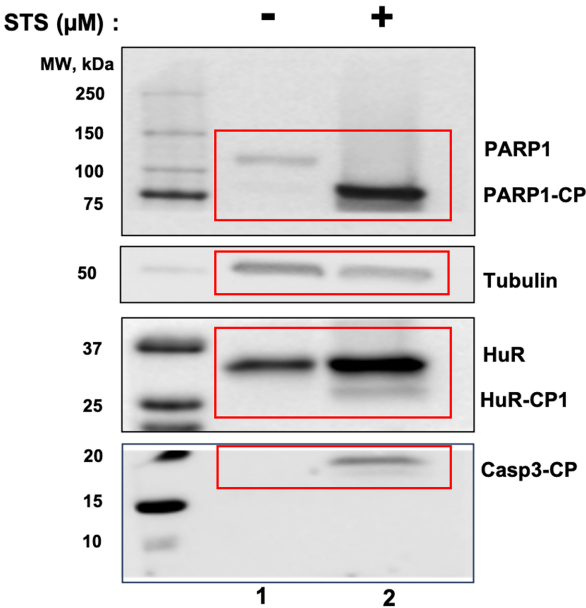

FigS1C

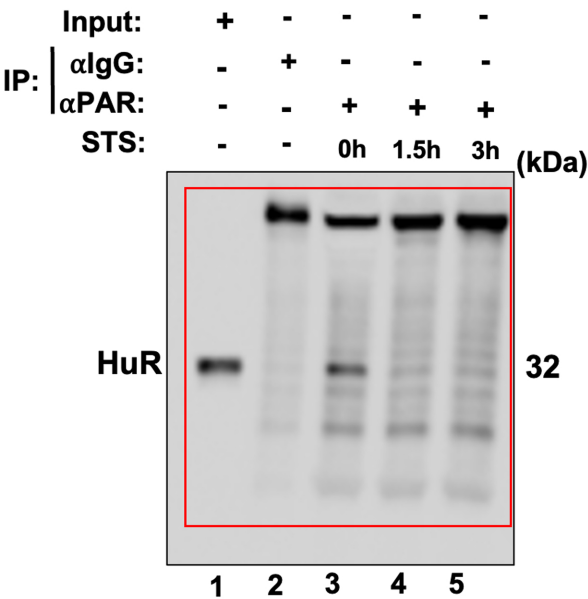

Supplement: Supplementary file 1 [file LSA-2023-02316_SdataFS1.pdf]

Fig1A

Representative blot for final figure

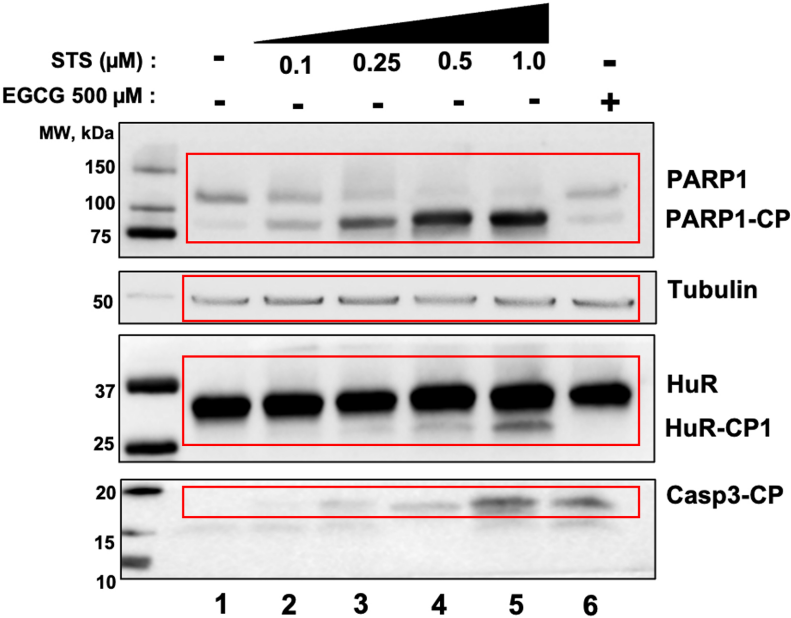

Fig1C

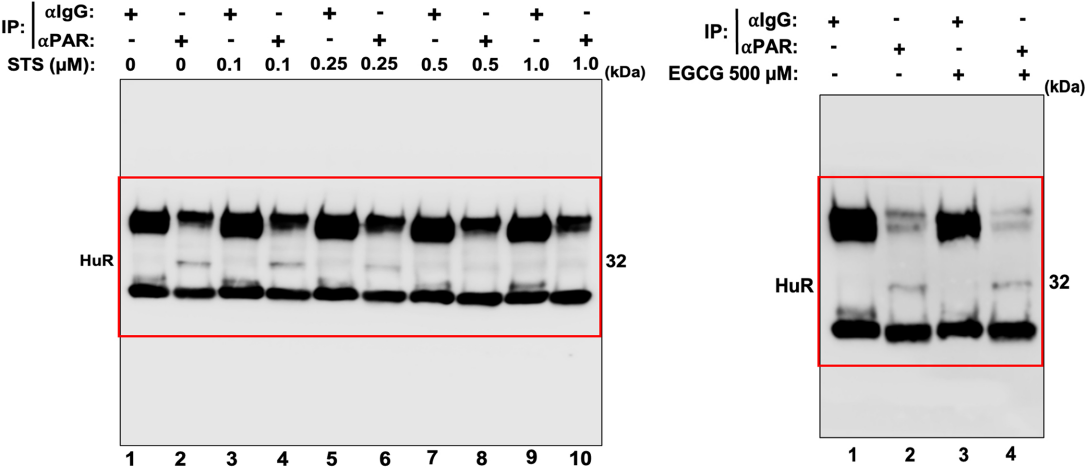

Supplement: Supplementary file 2 [file LSA-2023-02316_SdataF1.pdf]

FigS3B

Representative blot for final figure

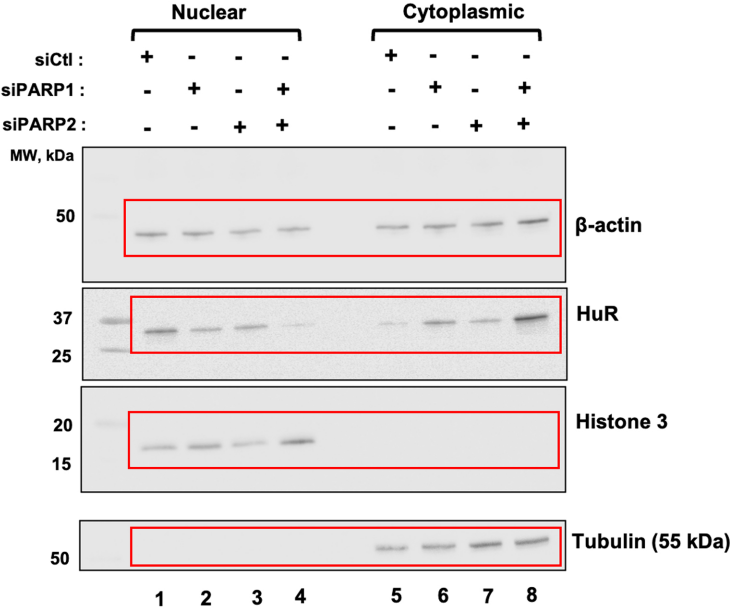

FigS3C

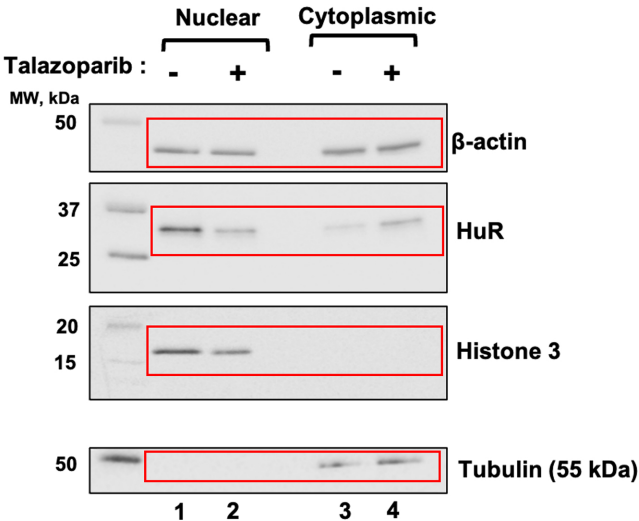

Supplement: Supplementary file 3 [file LSA-2023-02316_SdataFS3.pdf]

**Fig2C**

Representative blot for final figure

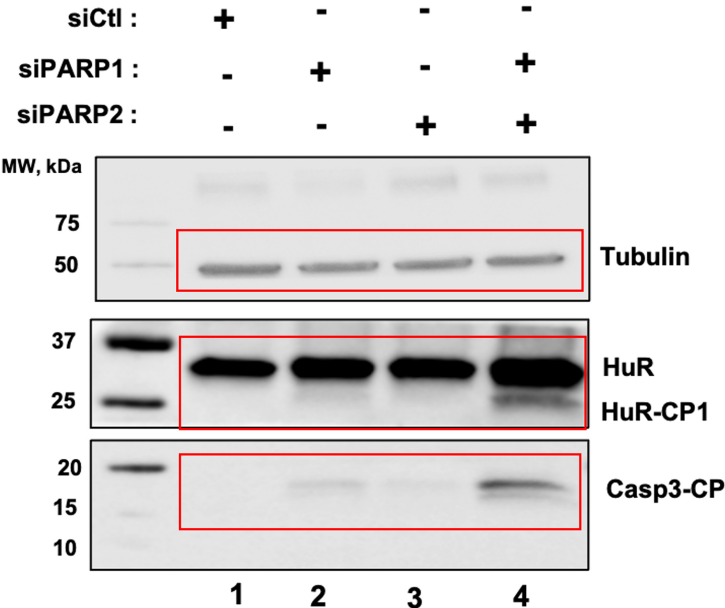

1

2

3

4

Supplement: Supplementary file 4 [file LSA-2023-02316_SdataF2.pdf]

**Fig3A**

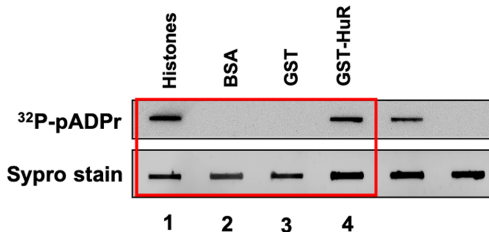

**Fig3C**

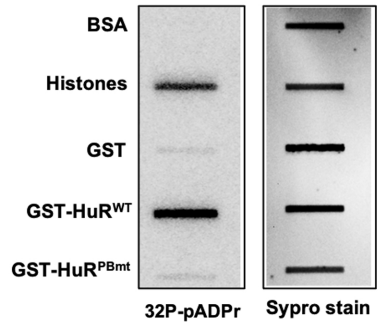

**Fig3D**

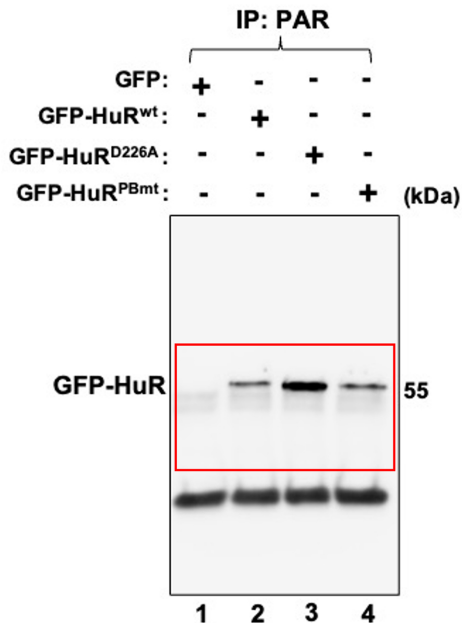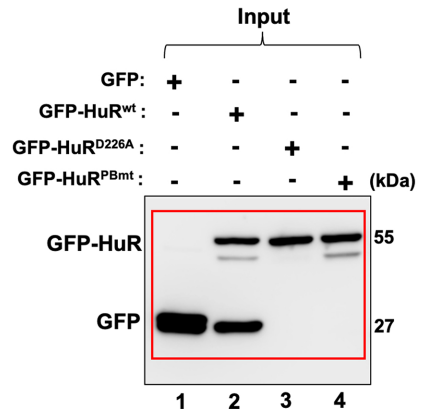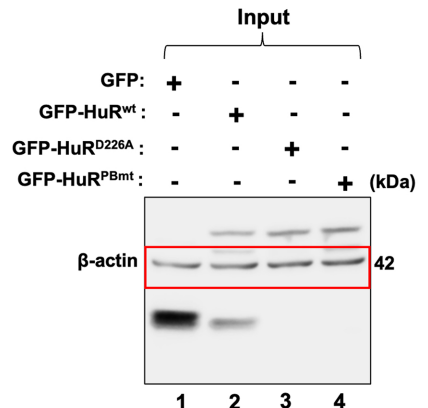

Supplement: Supplementary file 5 [file LSA-2023-02316_SdataF3.pdf]

FigS4

Representative blot for final figure

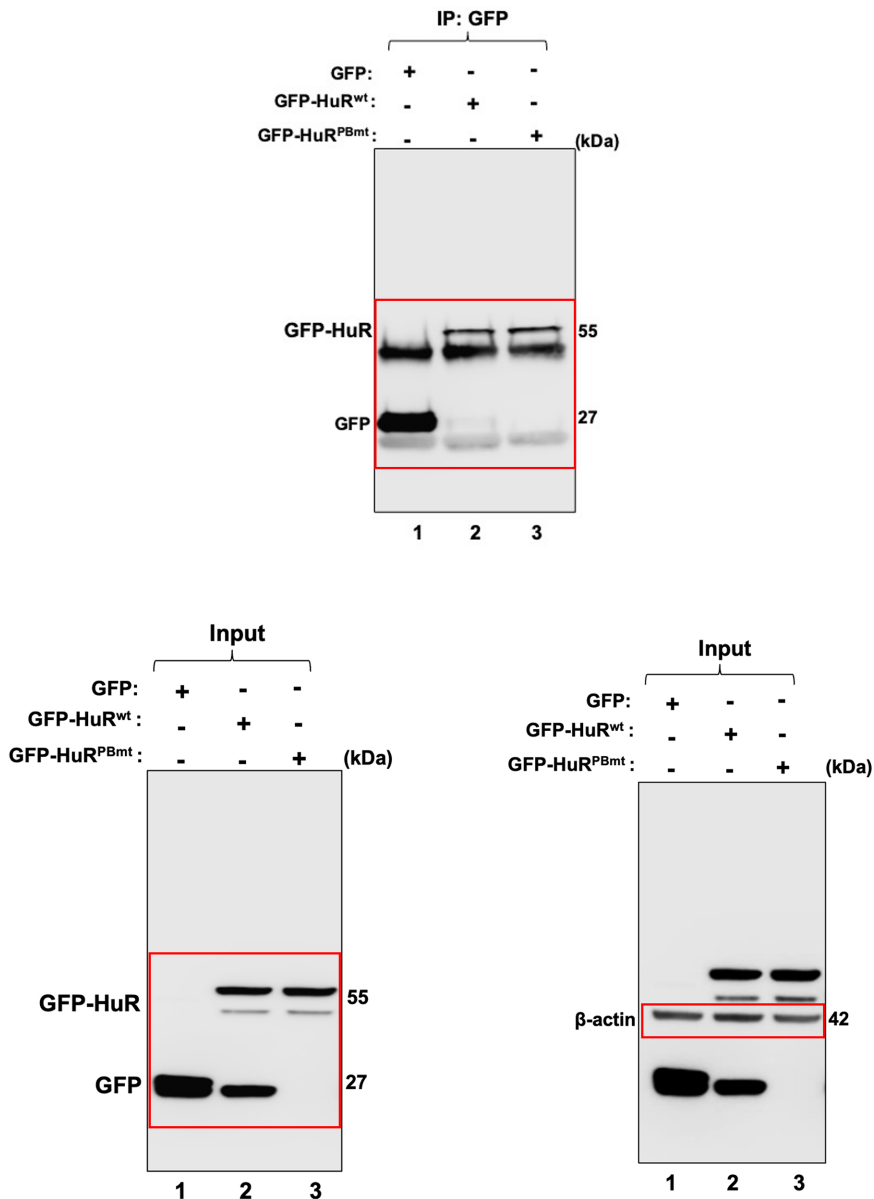

Supplement: Supplementary file 6 [file LSA-2023-02316_SdataFS4.pdf]

FigS5

Representative blot for final figure

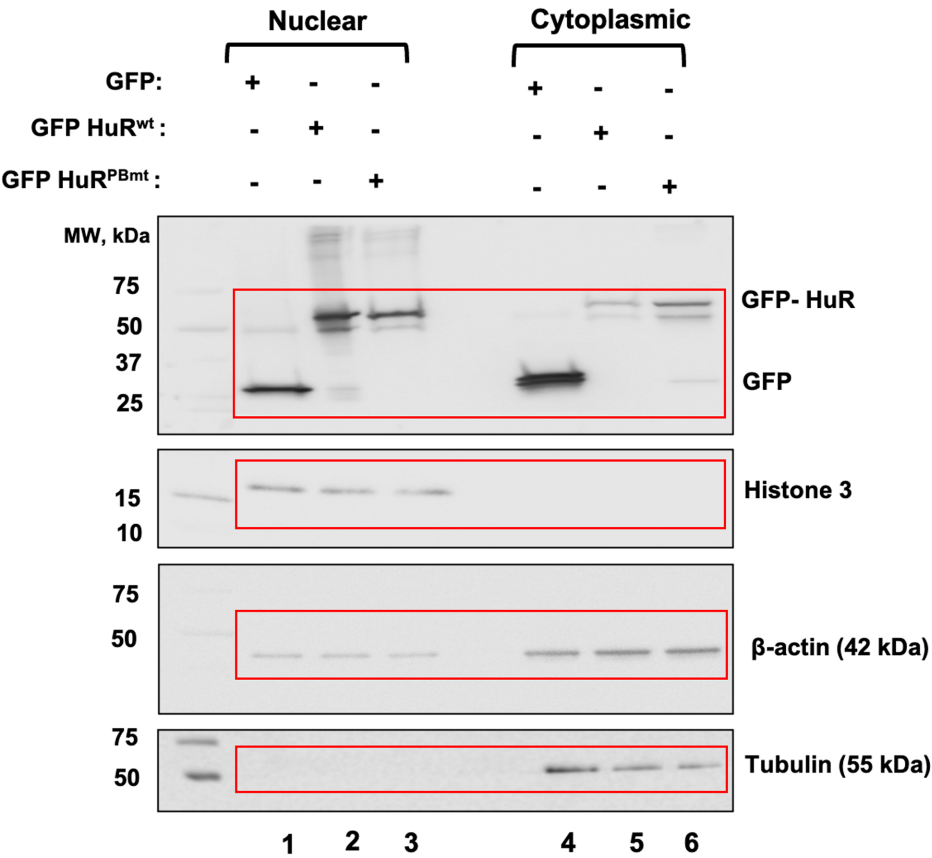

Supplement: Supplementary file 8 [file LSA-2023-02316_SdataFS5.pdf]

**Fig5A**

Representative blot for final figure

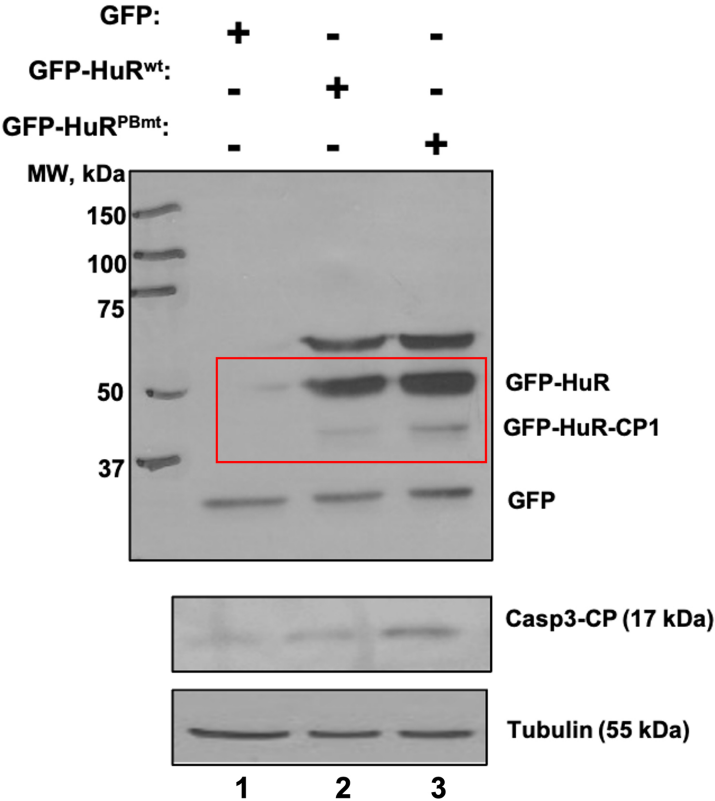

Supplement: Supplementary file 9 [file LSA-2023-02316_SdataF5.pdf]
