# Supplementary material for: pADP-ribosylation regulates the cytoplasmic localization, cleavage, and pro-apoptotic function of HuR
Source: Life Sci Alliance. 2024 Mar 27;7(6):e202302316. doi: 10.26508/lsa.202302316 (PMC10972696; doi:10.26508/lsa.202302316)

Fig4C

Representative blot for final figure

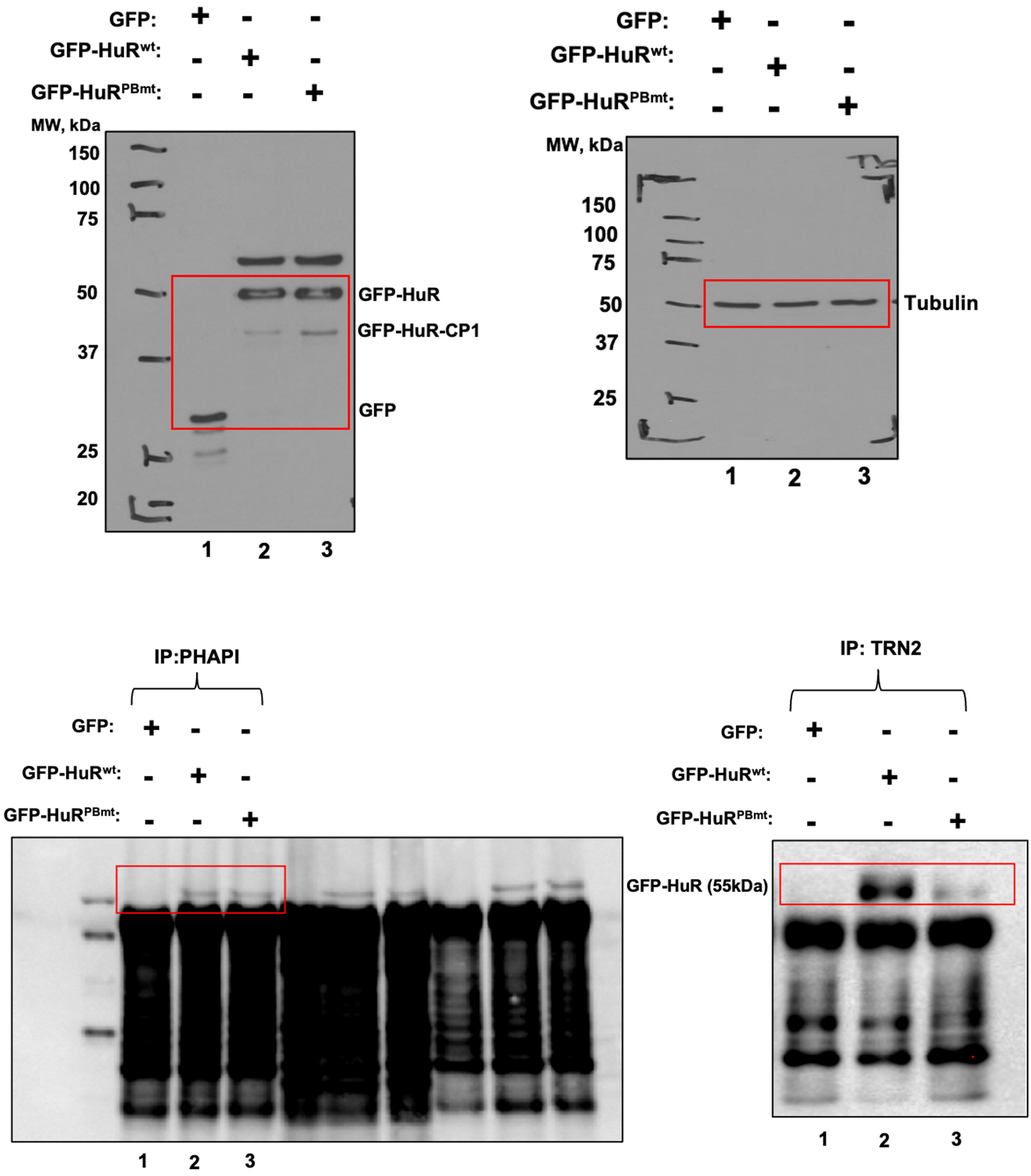

Supplement: Supplementary file 7 [file LSA-2023-02316_SdataF4.pdf]
